# Influence of the Anatomical Site on Adipose Tissue-Derived Stromal Cells’ Biological Profile and Osteogenic Potential in Companion Animals

**DOI:** 10.3390/vetsci10120673

**Published:** 2023-11-24

**Authors:** Carla Ferreira-Baptista, Rita Ferreira, Maria Helena Fernandes, Pedro Sousa Gomes, Bruno Colaço

**Affiliations:** 1 Centre for the Research and Technology of Agro-Environmental and Biological Sciences (CITAB), University of Trás-os-Montes e Alto Douro (UTAD), 5000-801 Vila Real, Portugal; al63939@alunos.utad.pt; 2 BoneLab—Laboratory for Bone Metabolism and Regeneration, Faculty of Dental Medicine, University of Porto, 4200-393 Porto, Portugal; mhfernandes@fmd.up.pt (M.H.F.); pgomes@fmd.up.pt (P.S.G.); 3 REQUIMTE/LAQV, University of Porto, 4100-007 Porto, Portugal; 4 REQUIMTE/LAQV, Department of Chemistry, University of Aveiro, 3810-193 Aveiro, Portugal; ritaferreira@ua.pt; 5 CECAV—Animal and Veterinary Research Centre UTAD, University of Trás-os-Montes and Alto Douro (UTAD), 5000-801 Vila Real, Portugal; 6 Associate Laboratory for Animal and Veterinary Sciences (AL4AnimalS), 5000-801 Vila Real, Portugal

**Keywords:** Mesenchymal stromal cells, adipose tissue, anatomical locations, osteogenic differentiation, companion animals

## Abstract

**Simple Summary:**

Adipose tissue-derived stromal cells (ADSCs) are of great interest in veterinary medicine, particularly in bone regeneration. These cells can be isolated from two anatomical regions—subcutaneous (SCAT) and visceral (VAT). However, depending on the anatomical region and animal species, ADSCs present different morphological and functional characteristics. Thus, the present work aimed to comprehensively review the different traits of ADSCs isolated from diverse anatomical sites in companion animals, i.e., dogs, cats, and horses, in terms of immunophenotype, morphology, proliferation, and osteogenic differentiation potential. The results show that the immunophenotype, proliferation, and osteogenic potential of ADSCs differ according to the tissue origin and the species. To ascertain the ideal source of ADSCs for each species, it is imperative to conduct studies that compare the profile of ADSCs isolated from various anatomical regions across different species. This selection is crucial for the effective administration of ADSCs at the site of the bone lesion.

**Abstract:**

Adipose tissue-derived stromal cells (ADSCs) have generated considerable interest in the field of veterinary medicine, particularly for their potential in therapeutic strategies focused on bone regeneration. These cells possess unique biological characteristics, including their regenerative capacity and their ability to produce bioactive molecules. However, it is crucial to recognize that the characteristics of ADSCs can vary depending on the animal species and the site from which they are derived, such as the subcutaneous and visceral regions (SCAT and VAT, respectively). Thus, the present work aimed to comprehensively review the different traits of ADSCs isolated from diverse anatomical sites in companion animals, i.e., dogs, cats, and horses, in terms of immunophenotype, morphology, proliferation, and osteogenic differentiation potential. The findings indicate that the immunophenotype, proliferation, and osteogenic potential of ADSCs differ according to tissue origin and species. Generally, the proliferation rate is higher in VAT-derived ADSCs in dogs and horses, whereas in cats, the proliferation rate appears to be similar in both cells isolated from SCAT and VAT regions. In terms of osteogenic differentiation potential, VAT-derived ADSCs demonstrate the highest capability in cats, whereas SCAT-derived ADSCs exhibit superior potential in horses. Interestingly, in dogs, VAT-derived cells appear to have greater potential than those isolated from SCAT. Within the VAT, ADSCs derived from the falciform ligament and omentum show increased osteogenic potential, compared to cells isolated from other anatomical locations. Consequently, considering these disparities, optimizing isolation protocols becomes pivotal, tailoring them to the specific target species and therapeutic aims, and judiciously selecting the anatomical site for ADSC isolation. This approach holds promise to enhance the efficacy of ADSCs-based bone regenerative therapies.

## 1. Introduction

Mesenchymal stromal cells (MSCs) are a population of multipotent cells [1,2], characterized by a high capacity for self-renewal [3,4] and a high proliferative rate when grown in culture at low density [2,5]. Beyond their proliferative prowess, MSCs exhibit an exceptional regenerative potential [6]. They actively secrete/produce a wide range of bioactive molecules, including cytokines and growth factors, as well as extracellular vesicles (EVs). These components serve pivotal roles in fostering trophic, paracrine, anti-inflammatory, and immunomodulatory responses, which promote the repair of damaged tissue and exhibit immunosuppressive attributes. Notably, MSCs possess the remarkable ability to gather on the sites of injury, facilitated by their capacity to migrate across the endothelium [7,8,9]. The beneficial biological characteristics of MSCs strongly indicate their vast potential in the realm of veterinary medicine, namely by paving the way for novel therapeutic strategies within regenerative medicine approaches, particularly in orthopedic treatments [10]. The prevalence of bone fractures among companion animals, including dogs, cats, and horses, is not only considerable but also holds great clinical implications.

To date, in the field of veterinary medicine, MSCs have been successfully isolated from various tissues, such as bone marrow [8,11,12,13,14,15,16,17,18], adipose tissue [13,14,15,16,19,20,21,22], umbilical cord/umbilical cord blood [14,18,21,22,23], synovial membrane [13,16,19,23], peripheral blood [8,24,25,26], placenta [21], endometrium [8], gingiva and periodontal ligament [27], and muscle and periosteum [15,20]. Among these options for sourcing MSCs, adipose tissue stands out as an exceptionally appealing reservoir due to its accessibility via low-risk techniques/minimally invasive approaches, further offering a substantial yield of MSCs [19].

ADSCs were isolated for the first time, in humans, in 2001 [28]. Nevertheless, in recent years, adipose tissue has emerged as the predominant and readily accessible cellular source for isolating MSCs in veterinary medicine [29,30]. Similar to human-derived ADSCs, those isolated from companion animals exhibit rapid in vitro expansion, enabling them to quickly attain the desired cell quantity for in vivo therapy. Additionally, they present significant osteogenic, adipogenic, and chondrogenic differentiation capabilities [5,31,32,33].

However, the features of MSCs may vary depending on the location of the harvest site, as there are differences between adipose tissue-derived stromal cells (ADSCs) originating from the subcutaneous and visceral region (SCAT and VAT, respectively) [34]. In addition, the animal species has been shown to influence the ADSCs profile [35]. Thus, the present work aims to comprehensively assess the distinct characteristics of ADSCs isolated from distinct anatomical locations in companion animals (i.e., cats, dogs, and horses). Our analysis will center on examining surface marker expression, cell proliferation rates, and potential for osteogenic differentiation.

## 2. Harvesting Location Influence on ADSCs’ Biological Profile

Adipose tissue can be obtained from two main deposits—subcutaneous (SCAT) and visceral (VAT)—each exhibiting different morphological and functional characteristics [36,37]. These differences have been extensively reported in numerous research studies over the years. In culture, ADSCs have been found to exhibit heterogeneous secretory profiles, as well as varying proliferative capabilities and microenvironmental signals, all influenced by their site of origin [38]. This underscores the importance of selecting the most suitable tissue site for ADSCs procurement, as it can significantly impact the yield, modify cellular traits, and ultimately influence differentiation potential.

In companion animals, there are numerous reports detailing ADSCs harvested from various locations (Table 1). Adipose tissue from the subcutaneous region is highly abundant [39,40] and readily accessible, due to its location beneath the skin. Consequently, it has emerged as the most extensively studied tissue in companion animals (Table 1); on the other hand, tissue from the visceral region is located around vital organs within the abdominal and thoracic cavities and can be collected from distinct locations [40]. Among companion animals, dogs have garnered extensive attention, with numerous studies focusing on characterizing ADSCs from the visceral region, a location that has received comparatively less attention in other species (Table 1).

The different profiles of ADSCs isolated from distinct anatomical locations within companion animals may translate into variations in therapeutic outcomes, underscoring the need for comprehensive investigation [35]. In this context, for each species (i.e., dogs, cats, and horses), ADSCs from different anatomical locations have been characterized, encompassing surface markers, proliferation, and osteogenic differentiation potential (Table 2 and Table 3). Below, we present a comprehensive review of the available insights concerning this matter, focusing on the most extensively studied companion animals, namely dogs, cats, and horses.

**Table 1:** Summary of reports detailing ADSCs harvested from various locations in different companion animals (dogs, cats, and horses).

	Anatomical Locations							
Species	Subcutaneous	Ligament Falciform	Omentum	Perirenal	Periovarian	Mesenteric	Retroperitoneal	Retrobulbar
**Dogs**	[9,13,35,41,42,43,44,45,46,47,48,49,50,51,52,53,54,55,56,57,58,59,60,61,62,63,64]	[44,48,57,61,65,66,67]	[5,41,42,55,56,68,69,70]	[55]	[48,64,65]	[71]	-	-
**Cats**	[35,72,73,74,75,76,77,78,79,80,81]	[82]	[83]	-	-	-	[79]	-
**Horses**	[6,11,15,40,47,84,85,86,87,88,89,90,91,92,93,94,95,96,97,98]	-	-	-	-	[11,90]	[98]	-

### 2.1. Dogs

ADSCs were first isolated in dogs from SCAT [99]. Since then, several studies have isolated and characterized ADSCs from various anatomical locations in the visceral region (VAT), namely falciform ligament, omentum, perirenal, periovarian, and mesenteric regions [5,41,42,44,48,55,56,57,61,64,65,66,67,68,69,70,71]. It is important to note that the morphological and functional characteristics of adipose tissue differ based on whether it originates from the SCAT or VAT regions [36,37]. To date, there have been no studies in dogs that compare the morphological characteristics of the tissues from the two anatomical regions; however, when samples from a similar region were studied, they exhibited identical characteristics in terms of mean adipocyte area and vascularization [65].

Primary characterization of canine ADSC cultures has predominantly focused on surface markers, morphology, and proliferation. However, the expression profile of ADSC surface markers is not unequivocally clear since it can fluctuate depending on the site of isolation. Numerous published studies have consistently indicated that ADSCs isolated from the subcutaneous region exhibit robust expression of CD90, CD44, CD29, and CD13, along with minimal or absent expression of CD54, CD34, CD45, MHC class II, CD11b, CD117, and CD3 (Table 2), which is in line with recognized standards for human ADSCs [46]. Nonetheless, CD105 and CD73, also acknowledged MSC markers highly expressed in human ADSCs, present a controversial expression in canine ADSCs [45,56]. Different authors have demonstrated both positive and negative expressions of CD73 and CD105 [45,49,51,54,55,56,57,60,90,100]. CD73, an ecto-5’-nucleotidase, is attached to the outer plasma membrane by a glycosylphosphatidylinositol (GPI) anchor and acts as a signal and adhesion molecule to support cells’ anchoring [55,101]. CD73 expression in SCAT may be related to donor age, with studies indicating elevated expression in young donors, whereas older donors exhibited diminished expression [90]. The expression of CD105, a high-affinity co-receptor for transforming growth factor (TGF)-β1 and TGF-β3, seems to vary depending on culture time and differentiation stage [35,55,102]. Regarding the VAT, the expression or absence of surface markers varies significantly based on the anatomical location of origin (Table 2). Nevertheless, most of the cultures established from different anatomical locations express the markers defined by the International Society for Cell & Gene Therapy (ISCT), i.e., CD105, CD73, and CD90. Notably, ADSCs isolated from mesenteric adipose tissue do not exhibit the surface marker CD73 [71]. Although CD73 is classified as an MSC marker by ISCT, its function in these cells remains relatively unexplored [101]. Recently, several studies have reported that CD73 is a regulatory factor for osteogenic differentiation of ADSCs, and its absence causes a lower bone mineral content in the bone [101,103]. The expression of CD73 is regulated by the Wnt/β-catenin signaling pathway, a key pathway in osteogenic activation [101]. This marker’s expression is also regulated by growth factors and cytokines, such as TGF-β, TNF-α, and IL-1β, which are present in the early phase of bone healing [101]. Thus, the absence of this surface marker in mesenteric-derived ADSCs may possibly interfere with their osteogenic capacity. Regarding ADSCs isolated from omentum adipose tissue, a consensus regarding the expression of CD90 is still lacking, as both positive and negative findings have been reported [35,42,55,56,68,69,70]. CD90 is a glycosylphosphatidylinositol (GPI)-linked membrane protein, which is expressed on the surfaces of hematopoietic stem cells, fibroblasts, peripheral T cells, epithelial cells, neurons, and thymocytes [104,105]. However, some studies have reported that CD90 expression tends to vary according to the differentiation state of cells in the osteoblast lineage. CD90 expression appears to increase in proliferating cells and decrease as calcified nodules form, reflecting the cells’ progress through the matrix maturation and mineralization stages [104,105]. Despite the reported differences in the expression of surface markers associated with several anatomical locations, studies have shown that regardless of the tissue of origin, ADSCs exhibit identical cytoskeletal organization, cellular arrangement, and morphology [35,42,52,56,65].

Another important aspect to take into consideration for broader clinical applications is the proliferation capability of ADSCs from different anatomical locations. Existing studies show variable proliferation patterns of ADSCs within the same individual, depending on the isolation site [106]. For instance, Neupane et al. reported that cells isolated from SCAT showed greater proliferative capacity than those isolated from VAT (omentum) [52]. Conversely, Bahamondes et al. observed no differences in proliferation potential between subcutaneous- and omentum-derived ADSCs [42]. Ferreira-Baptista et al., in a comparative study between two populations isolated from distinct VAT locations (falciform ligament- and periovarian-derived ADSCs), found that falciform ligament-derived ADSCs had lower proliferative capacity than periovarian-derived ADSCs [65]. Conversely, other studies have found an increased cell proliferation potential in VAT-derived populations, compared to those isolated from SCAT [72].

### 2.2. Cats

Very few studies have investigated the biological profile of ADSCs isolated from different anatomical locations in cats. Initial studies solely focused on the isolation and characterization of ADSCs from the subcutaneous region [75,77,78,82,83]. Only recently, ADSCs from the visceral region have been isolated and characterized [65,79]. The first study that compared the tissues of the two anatomical regions observed that VAT presented a higher average adipocyte area and more enhanced vascularity than SCAT [73]. Immunophenotyping of isolated ADSCs showed that, depending on the anatomical location, differences in the expression of surface markers were disclosed (Table 2). SCAT-derived cells exhibited high expression of CD90, CD44, CD29, CD105, CD73, MHC class I, and CD9, and minimal or absent CD34, CD45, MHC class II, CD14, and CD4 expression [74,76,77,78,80,81,82,83,107]. Conversely, cells from the different anatomical locations of VAT showed heightened expression of CD90, CD44, CD29 (falciform ligament), and CD105, while CD34, CD45, MHC class II, and CD14 expression was limited or absent [75,79].

An additional crucial factor to take into consideration involves the in vitro proliferation of ADSCs from distinct anatomical sites prior to their utilization. Although there are differences in the immunophenotype of ADSCs from different anatomical locations, Ferreira-Baptista et al. reported that cell populations isolated from the two anatomical regions—SCAT and VAT—exhibit similar proliferation rates [73]. However, a reported decrease in the proliferation rate of cat-derived ADSCs was observed after the fifth passage [20,89]. Interestingly, Lee et al. also demonstrated a decreased expression of surface markers across passages [20].

### 2.3. Horses

Although the first study on equine-derived ADSCs was focused on cells isolated from the subcutaneous region [98], currently, several studies have characterized ADSCs from various anatomical locations within the visceral region, namely the retroperitoneal and mesenteric adipose depots [11,84]. To date, no comprehensive research has been conducted to compare the adipose tissue morphology and organization between the two distinct anatomical regions in horses. However, the morphology of equine ADSCs from different anatomical locations is described as typical for MSCs, with the cells exhibiting a fibroblast-like morphology and well-defined F-actin cytoskeleton extending across the entire cytoplasm [84,91,108]. Although equine-derived ADSCs’ characterization follows the same principles as MSCs of human origin, several studies have shown that the expression of surface markers in equine ADSCs follows a different pattern (Table 2) [29,93]. SCAT-derived populations show high expression of CD44, CD29, CD117, and MHC class I, yet low or no expression of CD54, CD34, CD45, MHC class II, CD11b, and CD79α. Concerning CD90, CD105, and CD73, several authors have reported both positive and negative expression of these markers (Table 2). This variability can be explained by multiple factors, including differences in culture conditions and individual differences between donors [109]. Despite the often consistent expression profile among equine-derived ADSCs from different donors, inherent individual variations lead to a heterogeneous expression pattern, even within identical anatomical sites of harvest [93]. Regarding VAT-derived populations, the limited available studies report high expression of CD90, CD44, and CD105, and low or no expression of CD45, MHC class II, CD11b, and CD79α [11,90,98]. Furthermore, there are reports indicating that cell proliferation of VAT-derived ADSCs is higher than that of those isolated from SCAT [84].

**Table 2:** ADSC surface marker expression harvested from different anatomical locations in dogs, cats, and horses. (+ present; − absent; +/− present and absent).

Source	CD Markers																			References
	90	44	29	105	13	54	133	73	34	45	MHC Class II	11b	117	31	14	MHC Class I	9	79α	4	
**Dogs**																				
**Subcutaneous**	+	+	+	+/−	+	−		+/−	−	−	−	−	−	−						[9,41,42,44,45,46,49,51,54,55,56,57,60,63,64,70]
**Falciform Ligament**	+	+	+	+		+			−	−	−				−					[45,61,65,66,67]
**Omentum**	+/−	+	+	+	+		+	+	−	−	−	−		+/−	−					[5,42,55,64,68,69,70]
**Perirenal**	+			+				+		−										[57]
**Periovarian**	+	+		+					−	−										[45]
**Mesenteric**	+		+					−	−	−										[71]
**Cats**																				
**Subcutaneous**	+	+	+	+				+	−	−	−				−	+	+		−	[72,73,74,75,76,77,78,80,81]
**Omentum**	+	+		+					−	−					−					[78]
**Falciform Ligament**	+	+	+	+					−	−	−				−					[79]
**Horses**																				
**Subcutaneous**	+/−	+	+	+/−	−			+/−	−	−	−	−	+			+		−		[6,11,47,52,84,86,88,90,91,92,93,98,110]
**Retroperitoneal**	+	+		+						−	−									[98]
**Mesenteric**	+			+						−	−	−						−		[11,90]

**Table 3:** Osteogenic potential on ADSCs yielded from different anatomical locations in companion animals.

Osteogenic Potential		Source											
		Dogs					Cats			Horse			
		Subcutaneous	Omentum	Falciform Ligament	Periovarian	Perirenal	Subcutaneous	Retroperitoneal	Omentum	Subcutaneous	Retroperitoneal	Retro- Bulbar	Mesenteric
**MINERALIZATION ASSAY**	**Alizarin Red**	High mineralization matrix [13,42,53,55,56,58,59,63,64]	High mineralization matrix [42,55,56]	High mineralization matrix [59]	Low mineralization matrix [64]	High mineralization matrix [55]	High mineralization matrix [35,75]	-	High mineralization matrix [83]	High mineralization matrix [11,86,89,92,95,98]	Low mineralization matrix [98]	-	High mineralization matrix [11]
	**von Kossa**	High staining [47,54,61,99]	-	-	-	-	-	-	-	High staining [15,88,91,94,96,97]	-	Low staining [96]	-
**GENE EXPRESSION**	**Osteogenic markers**	High expression of RUNX2, COL1A1, SPP1, ALP, SP7, BGLAP BMP7 and BSP [50,52,53,55,56,63,99] Low expression of BGLAP [56]	High expression of RUNX2, COL1A1 and BGLAP [55,56]	High expression of SOX9, RUNX2, COL1A1 and SP7 [65]	Low expression of SOX9, COL1A1, SP7 and BGLAP [65]	High expression of SP7 and BGLAP [55]	-	-	-	High expression of RUNX2, BGLAP, ALP, SP7, COL1A1 and SPP1 [15,85,91,92,95,97]	-	-	-
**BIOCHEMICAL TECHNIQUES**	**ALP activity**	Low levels of ALP activity [55] High levels of ALP activity [47,50,52]	High levels of ALP activity [55]	-	-	Low levels of ALP activity [55]	Low levels of ALP activity [79]	High levels of ALP activity [79]	-	High levels of ALP activity [43,85,89,96,98]	High levels of ALP activity [98]	Low levels of ALP activity [96]	-
**IMMUNO-EXPRESSION**	**Osteopontin**	High expression [55]	High expression [55]	-	-	High expression [55]	-	-	-	-	-	-	-
**CYTOCHEMICAL TECHNIQUES**	**Collagen**	High staining [52]	-	High staining [65]	High staining [65]	-	-	-	-	-	-	-	-
	**Alkaline Phosphatase**	High staining [43]	-	High staining [65]	High staining [65]	-	Low staining [79]	High staining [79]	-	High staining [15,89,94,97]	-	-	-

## 3. Harvesting Location Influence on ADSC’s Osteogenic Differentiation

ADSCs are unevenly distributed in tissues, giving rise to location-dependent variations in the proliferative potential and differentiation capabilities of cells from the same individual. Several studies have reported that the osteogenic potential of ADSCs may vary depending on the adipose tissue harvesting site [52,106]. However, despite recent efforts to characterize ADSCs based on their origin, the issue of harvesting location is still a challenge. While specific anatomical locations have been extensively studied in certain companion animals, there is still a dearth of foundational knowledge to bolster our comprehension of ADSCs’ osteogenic potential. Therefore, in the context of advancing bone regeneration strategies, it is important to select the optimal ADSC tissue sources for each unique species. Most studies on this topic have primarily focused on dogs, with fewer investigations conducted on cats and horses.

The osteogenic induction of ADSCs in most studies has centered on the use of the combination of dexamethasone, β-glycerophosphate, and ascorbic acid. However, recent studies in dogs and cats have also used retinoic acid (RA) as an osteogenic inducer [65,73,111]. Dexamethasone is a synthetic glucocorticoid that promotes increased expression of osteogenic markers by the expression of bone markers, such as RUNX family transcription factor 2 (RUNX2), alkaline phosphatase (ALP), osterix (SP7) osteocalcin (BGLAP), and osteopontin (SPP1) [112,113,114,115]; β-glycerophosphate induces calcification and mineralization of the extracellular matrix [112,115]; ascorbic acid modulates extracellular matrix characteristics, promoting an increase in osteogenic differentiation [112,115]; and RA increases the expression of osteogenic transcription factors and increases the mineralization of the extracellular matrix and bone nodule deposition, promoting osteogenic differentiation [62,116,117].

Regarding the investigations scrutinized in this review, after induction, osteogenic differentiation was mainly assessed by ALP activity, gene, and protein expression of osteogenic markers, cytochemical staining of collagen and/or alkaline phosphatase, and mineralization assays (cytochemical methodologies with Alizarin Red S and/or von Kossa stains) (Table 3).

### 3.1. Dogs

To date, several studies have explored the osteogenic potential of dog ADSCs, from various anatomical locations. A thorough examination of Table 3 reveals discernible differences in osteogenic potential depending on the anatomical location. Cultures isolated from subcutaneous, omentum, falciform ligament, and perirenal adipose tissue seem to produce a more mineralized matrix compared to cultures derived from the periovarian-derived ADSCs [13,35,42,53,55,56,58,59,63,64,118]. In accordance, when osteogenic markers were analyzed, the expression of early markers (RUNX2 and collagen type I alpha 1 chain (COL1A1)) and late markers (SP7 and BGLAP) was higher in subcutaneous-, omentum-, falciform ligament-, and perirenal-derived ADSCs, than in periovarian-derived ADSCs [5,35,50,53,55,56,63,65,99,118]. Intriguingly, although ADSCs isolated from the subcutaneous region expressed BGLAP, cultures isolated from VAT, particularly from the omentum and perirenal regions, exhibited a higher expression [55,56].

ALP activity emerges as a relatively ubiquitous assay among studies, consistently observed in ADSC cultures isolated from the subcutaneous, omentum, and perirenal regions. ALP is an early marker of osteoblastic differentiation that plays an important role in the matrix mineralization process, reducing pyrophosphatase—an inhibitor of mineral formation—to produce inorganic phosphate and free calcium [119,120,121]. A comparative study encompassing cells from three anatomical locations revealed that those derived from the omentum exhibit higher ALP activity than their counterparts [35,55].

In terms of cytochemical analysis, while the staining intensity of collagen and alkaline phosphatase increases over time in cultures established from all anatomical locations [43,47,60,61,65,99,106], a comparative study by Ferreira-Baptista et al. revealed higher staining in falciform ligament-derived ADSCs compared to periovarian-derived ADSCs [65].

In summation, although various anatomical locations have been explored, ADSCs isolated from omentum showed greater expression of osteogenic markers, such as RUNX2, COL1A1, and BGLAP, and an increased ALP activity, as compared to those isolated from perirenal and subcutaneous regions [55,56]. In a comparative analysis of two VAT cultures, falciform ligament-derived ADSCs showed increased osteogenic markers expression, such as SRY-Box transcription factor 9 (SOX9), RUNX2, COL1A1, and SP7, and cytochemical staining of collagen compared to periovarian-derived ADSCs [65].

### 3.2. Cats

The osteogenic potential of cat ADSCs varies depending on the anatomical region. Until now, osteogenic studies have been exclusively conducted on subcutaneous, retroperitoneal, and omentum-derived ADSCs. Among these, the subcutaneous region has been the most extensively investigated (Table 3). In SCAT, multiple studies have reported highly mineralized matrix levels yet low ALP activity, both within biochemical and cytochemical assessments [35,73,77]. While a few studies have explored various anatomical locations [35,75,83], only Ferreira-Baptista et al. [79] conducted a comparative analysis of the osteogenic potential of SCAT-derived and VAT-derived ADSCs. Upon this comparison, cultures derived from VAT exhibited higher ALP activity and cytochemical staining, demonstrating a superior osteogenic potential compared to SCAT-derived cultures [73].

### 3.3. Horses

In recent years, the potential of equine-derived ADSCs has also been explored in the field of orthopedics. Most of the studies have isolated and characterized ADSCs from the subcutaneous region, possibly by the ease of access this tissue affords. Subcutaneous-derived ADSCs show a high mineralized matrix in culture, a high expression of osteogenic markers, high levels of ALP activity, and substantial cytochemical staining of ALP and mineral deposits [11,15,84,85,86,87,88,92,94,95,96,97,98,108,110]. So far, only a limited number of studies have explored the visceral region, with the majority focusing on the comparative analysis of osteogenic differentiation between cells isolated from the two regions [11,84,96]. Arnhold et al. demonstrated that although cells from both anatomical regions—SCAT and VAT (retroperitoneal)—exhibited high levels of ALP activity in culture, only SCAT-derived populations presented extracellular matrix mineralization, indicative of calcium deposition [84]. In another comparative study involving subcutaneous- and retrobulbar-derived ADSCs, it was also reported that subcutaneous-derived populations showed higher ALP activity and extracellular calcium phosphate deposits in contrast to their retrobulbar-derived counterparts [96].

To date, the analysis of the osteogenic differentiation of equine-derived ADSCs from the two anatomical regions—SCAT and VAT—revealed that SCAT-derived cells showed superior osteogenic capacity to those from VAT [84,96]. However, additional osteogenic studies conducted on ADSCs isolated from the visceral region will be required to validate these results.

## 4. Conclusions and Future Perspectives

This comprehensive review underscores the necessity for studies comparing the profile of ADSCs isolated from different anatomical regions, across different species, for prospective clinical applications in the frame of veterinary medicine. This will enhance our understanding of the most suitable tissue site for effective implementation in bone tissue repair and regeneration. To date, canine ADSCs have been isolated and characterized for surface markers, morphology, proliferation, and osteogenic differentiation from various anatomical locations. Conversely, in the case of cats and horses, ADSC isolation and characterization have been limited to a few anatomical locations.

**Figure 1:**
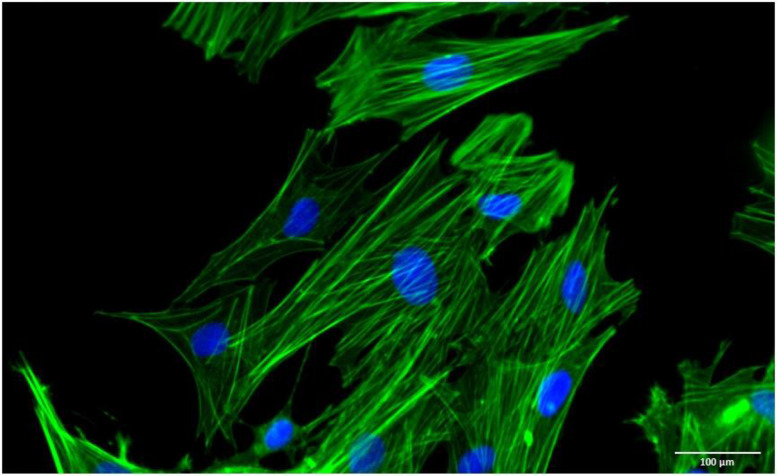
Representative image of the morphology. Filaments of F-actin = green; and nucleus = blue.

Overall, it is known that the criteria for identifying ADSCs proposed by ISCT and defined for human cell populations cannot be applied to veterinary medicine. ADSCs obtained from animals do exhibit adherence to cell culture surfaces under conventional culture conditions and possess multilineage differentiation potential. However, immunophenotyping demonstrates variability contingent upon the collection site and species. Although there are some differences between species in the expression and absence of surface markers, the majority of ADSCs from diverse anatomical locations in dogs, cats, and horses expressed CD90, CD44, CD29, and CD105 and presented low or no expression of CD34, CD45, and MHC class II. Despite these differences, ADSCs, regardless of tissue of origin and species, exhibit a fibroblast-like arrangement with an elongated morphology and well-defined F-actin cytoskeleton (Figure 1), characteristics also described for human cells [35,42,52,56,65,73,122,123,124,125]. Comparatively, the proliferation potential tends to be higher in VAT-derived ADSCs for both dogs and horses, as compared to their subcutaneous counterparts [72]. The proliferation in ADSCs of different anatomical locations in cats is still poorly studied, but from what is known, tends to be similar [73].

Delving into the osteogenic differentiation potential, in dogs, VAT-derived ADSCs appear to have higher potential than SCAT-derived ADSCs. Within the VAT, ADSCs derived from the falciform ligament and omentum show increased expression compared to other anatomical locations. In cats, VAT-derived ADSCs also show a higher capacity, while SCAT-derived ADSCs show higher potential in horses. Given the variability in proliferation and differentiation potential based on tissue origin and species, it becomes imperative to refine protocols for the purpose of strategically selecting the ideal ADSC tissue source for each species, ultimately enhancing cell-based orthopedic therapies.

The escalating prevalence of bone fractures within the field of veterinary medicine has seen a rise in recent years, underscoring the necessity for novel regenerative medicine approaches, either cell-based or cell-free therapies, that facilitate bone regeneration in scenarios where conventional healing is compromised [126,127]. While clinical studies involving the administration of ADSCs at fracture sites remain relatively sparse in veterinary medicine, some researchers report that the factors secreted by ADSCs may have great advantages compared to cell-based therapies [128,129]. The factors secreted by ADSCs can be manufactured, freeze-dried, packaged, and transported more easily [130,131,132], thus avoiding the problems related to the use of fresh or frozen ADSCs, since the cell viability of these cells after cryopreservation is controversial [133,134]. Although there are studies that report that cryopreservation can alter the structure and function of cells, there are others that report that the morphology and proliferation of ADSCs are maintained after cryopreservation [134,135].

Recent studies have changed the view of ADSCs, drawing attention to an array of bioactive factors that are secreted by ADSCs, which may play an important role in bone fracture repair [132]. Therefore, beyond identifying the ideal ADSC source for each species, administering ADSCs directly at the fracture site becomes even more important in new cell-free therapies.

## Data Availability

Not applicable.
